# Reduced blood EPAC1 protein levels as a marker of severe coronary artery disease: the role of hypoxic foam cell-transformed smooth muscle cells

**DOI:** 10.1186/s12967-025-06513-3

**Published:** 2025-05-09

**Authors:** Eduardo Garcia, Lene Claudi, Maria Teresa La Chica Lhoëst, Anna Polishchuk, Valerie Samouillan, Aleyda Benitez Amaro, Janet Pinero, Joan Carles Escolà-Gil, Eduard Sabidó, Ruben Leta, David Vilades, Vicenta Llorente Cortes

**Affiliations:** 1 Institute of Biomedical Research of Barcelona (IIBB), Spanish National Research Council (CSIC), Barcelona, Spain; 2 Institut de Recerca Sant Pau (IR SANT PAU), Sant Quintí 77–79, 08041 Barcelona, Spain; 3 Departament de Medicina, Universitat Autònoma de Barcelona, 08041 Barcelona, Spain; 4 Departament de Bioquímica i Biologia Molecular, Universitat Autònoma de Barcelona, 08041 Barcelona, Spain; 5 CIRIMAT, Université de Toulouse, Université Paul Sabatier, Equipe PHYPOL, 31062 Toulouse, France; 6 Research Programme on Biomedical Informatics (GRIB), Department of Experimental and Health Sciences (DCEXS), Hospital del Mar Medical Research Institute (IMIM), Universitat Pompeu Fabra (UPF), Barcelona, Spain; 7 CIBER de Diabetes y Enfermedades Metabólicas Asociadas (CIBERDEM), 28029 Madrid, Spain; 8 Proteomics Unit, Centre de Regulació Genòmica, Barcelona Institute of Science and Technology; Universitat Pompeu i Fabra (UPF), Barcelona, Spain; 9 Cardiac Imaging Unit, Department of Cardiology, Hospital de la Santa Creu i Sant Pau, Universitat Autonoma de Barcelona, Barcelona, Spain; 10 CIBER de Enfermedades Cardiovasculares CIBERCV, Institute of Health Carlos III, 28029, Madrid, Spain

**Keywords:** Coronary artery disease (CAD), Coronary computed tomographic angiography (CCTA), Human coronary vascular smooth muscle cells (hcVSMC), EPAC1, Biomarker

## Abstract

**Background:**

Vascular smooth muscle cells loaded with cholesterol (foam-VSMCs) play a crucial role in the progression of human atherosclerosis. Exchange Protein Directly Activated by cAMP 1 (EPAC1) is a critical protein in the regulation of vascular tone, endothelial function, and inflammation. Our objectives were to identify proteins specifically secreted by foam human coronary VSMCs (foam-hcVSMC) to evaluate their potential as circulating biomarkers for diagnosing coronary artery disease (CAD), and to ascertain the mechanisms underlying their levels in the blood of patients with CAD.

**Methods and results:**

Differential proteomics identified EPAC1 as a differential foam-hcVSMC-secreted protein. Circulating EPAC1 levels were measured by ELISA in blood from 202 patients with suspected CAD who underwent coronary computed tomography angiography (CCTA). Blood EPAC1 levels were significantly lower in CAD patients compared to controls (p < 0.001). EPAC1 levels were reduced in both men and women with severe CAD (SIS > 4) compared to those with moderate CAD (SIS 1–4). ROC analysis identified 9.16 ng/ml as the optimal EPAC1 cut-off for severe CAD. At this threshold, EPAC1 predicted severe CAD (SIS > 4) with 69.6% sensitivity and 79.4% specificity, outperforming hs-CRP and hs-TnT in predicting CAD severity. Real-time PCR and Western blot analysis revealed that human foam-SMCs under hypoxic conditions exhibited a significant reduction in EPAC1 mRNA (p = 0.013) and protein (p < 0.001) levels.

**Conclusions:**

These findings suggest that circulating EPAC1 protein levels lower than 9.16 ng/mL are predictive of severe CAD in humans. Hypoxic foam-SMCs, characteristic of advanced atherosclerotic lesions, exhibit diminished production of EPAC1, potentially contributing to the decreased circulating EPAC1 levels in patients with severe CAD.

**Supplementary Information:**

The online version contains supplementary material available at 10.1186/s12967-025-06513-3.

## Introduction

Coronary artery disease (CAD) remains the leading cause of morbidity and mortality worldwide, accounting for approximately 30% of deaths attributed to cardiovascular disease. Major contributors to the development of atherosclerosis and atherothrombosis include smoking, high blood pressure, low-density lipoprotein (LDL) cholesterol, and diabetes [1]. However, many individuals experience cardiovascular events despite the absence of these traditional risk factors.

A recent publication reported that 60% (84 candidate causal genes) of known CAD-associated variants identified through genome-wide association studies (GWAS) show statistically significant expression quantitative trait locus (eQTL) or splicing QTL effects in vascular smooth muscle cells (SMCs). Another study found that 20% of eQTLs identifying candidate genes for GWAS loci are specific to quiescent SMCs, while 355 are specific to proliferating SMCs. These findings suggest that arterial SMCs, the most abundant cell type in arterial lesions, in particular in certain prothrombotic phenotypic states such as foam SMC, could contribute significantly to total CAD risk. Recent research using macrophage-specific cell markers, SMC lineage-tracing mice, and GWAS CAD loci further highlights the substantial role of SMCs in determining lesion progression and CAD risk [2–4].

Several research groups, including ours, previously highlighted the critical role of vascular SMCs loaded with cholesterol (foam-SMCs) in the progression of human atherosclerosis and their association with both acute and chronic cardiovascular events [5–7]. Notably, over 40% of foam cells in atherosclerotic lesions are derived from SMCs [8–10]. LDL aggregates, which bind to the LRP1 receptor, represent a key driver of the phenotypic transformation of human coronary VSMCs (hcVSMC) into foam—prothrombotic hcVSMCs with high pathogenic potential [5]. The susceptibility of LDL to aggregation [11, 12] and the levels of circulating LRP1 [13, 14] are both associated with an increased risk of future cardiovascular events in general population.

SMCs exhibit a robust capacity to secrete microvesicles that carry proteins involved in vascular calcification and coagulation pathways [15–18]. Vesicles derived from atherosclerotic plaques exhibit high thrombogenicity due to their enrichment in tissue factor (TF), a key initiator of the coagulation cascade [18]. The release of TF-enriched vesicles by vascular cells appears to be stimulated by factors such as platelet-derived growth factor (PDGF), tumor necrosis factor-α (TNFα) [19, 20], and modified lipoproteins [5, 21, 22]. These findings highlight the critical role of foam SMC-derived microvesicles and their protein cargo in driving atherosclerotic plaque progression. Furthermore, these earlier findings indicate that the SMC secretome, particularly that derived from foam-SMCs, holds substantial potential for identifying specific biomarkers associated with the progression of atherosclerotic plaques and CAD.

Another crucial factor and a strong predictor of CAD progression is neovascularization of the vasa vasorum. A powerful driver of neovascularization is intraplaque hypoxia [23–25], which increases local oxygen consumption by foam cells and exacerbates the process of foam cell formation through the overexpression of specific lipoprotein receptors under these oxygen-deficient conditions [26–28]. These findings suggest that foam-SMCs generated under hypoxic conditions exhibit a phenotype characteristic of advanced disease stages, with potential as a source of innovative biomarkers of intraplaque hypoxia and severe CAD.

An interesting protein modulated by hypoxia is the exchange protein activated by cAMP isoform 1 (EPAC1) [29, 30]. EPAC proteins comprise at least two cAMP-GEF proteins isoforms EPAC1 and EPAC2. They act as critical sensors of cyclic AMP and function as a guanine nucleotide exchange factor (GEF) that activates Rap1 independently of protein kinase A (PKA), representing an alternative cAMP-mediated signaling pathway [31]. In particular, EPAC1 is encoded by RAPGEF3 gene in humans and is expressed in heart, blood vessels and kidney, playing a critical role in the regulation of vascular tone, endothelial function, and inflammation [32]. EPAC1 maintains the endothelial barrier under both physiological and pathological conditions [33], and blocks the increase in endothelial permeability induced by pro-inflammatory mediators such as thrombin [34]. Unlike the clear protective role of EPAC1 in endothelial cells, EPAC1 appears to promote foam cell formation in macrophages since EPAC1 inhibition reduces the formation of macrophage foam cells and their associated proinflammatory effects, contributing to the attenuation of atherosclerosis in a triple knockout mouse model [35]. In the case of SMC, the role of EPAC1 activity in the vascular wall remains controversial, with conflicting results regarding its protective or pathological effects. On one hand, EPAC1 deficiency has been shown to inhibit neointima formation in Epac1(-/-) mice [36], and a specific EPAC1 inhibitor blocks SMC migration, inhibiting pathological angiogenesis in mice [37]. On the other hand, EPAC1 activation has been shown to play a role in the beneficial effects of prostaglandin D2 receptor 1 (DP1) by preventing the phenotypic switch of vascular smooth muscle cells to myofibroblasts [38].

EPAC1 is regulated in cardiac muscle under stress conditions and contributes to the development of cardiac dysfunction [39, 40]. EPAC1-deficient mice are protected against cardiac fibrosis and hypertrophy induced by chronic activation of the beta-adrenergic receptor (β-AR) [41], and age [42]. Moreover, the spontaneous calcium release from the SR is a process attenuated in EPAC-deficient mice [43, 44]. In line with these results, treatment of mice with a selective EPAC1 inhibitor, CE3 F4, prevents atrial and ventricular arrhythmias [45]. In addition, EPAC contributes to cardiac rhythm disorders by increasing the expression of pro-arrhythmic channels such as TRPC3/4 (Transient Receptor Potential Canonical) in a porcine experimental model [46], and rat ventricular cardiomyocytes [46].

High-sensitivity troponin T (hsTnT) and high-sensitivity C-reactive protein (hsCRP) are widely regarded as essential biomarkers for assessing cardiovascular risk and the early detection of cardiovascular diseases [47, 48]. HsTnT is particularly effective in detecting myocardial injury, while hsCRP serves as an indicator of vascular inflammation, making both biomarkers valuable predictors of adverse cardiovascular events [48]. High-sensitivity troponin T (hsTnT) is a cardiac muscle protein released into the bloodstream during myocardial injury, even in the early stages, allowing for the early detection of myocardial infarction and the assessment of subclinical damage in patients with heart failure or hypertension [49]. In contrast, high-sensitivity C-reactive protein (hsCRP), a biomarker of systemic inflammation strongly associated with the development and progression of atherosclerosis, is valuable for evaluating cardiovascular risk in individuals without established disease and monitoring responses to anti-inflammatory or lipid-lowering treatments, such as statins [50, 51]. Although hs-TnT and hs-CRP have consistently been shown to be useful markers of myocardial damage and inflammation, respectively, they exhibit limited associations with atherosclerotic plaque burden and acute coronary events [52]. Therefore, there is a clinical need to identify new blood biomarkers that can predict atherosclerotic plaque burden in patients with suspected ischemic heart disease.

Building on these previous findings, our objectives are to: (1) identify proteins differentially secreted by foam-hcVSMCs, (2) assess the potential of these differentially secreted proteins as circulating biomarkers for the diagnosis of CAD, and (3) investigate the mechanisms influencing the levels of foam-hcVSMC-secreted proteins in the blood of CAD patients.

## Material and methods

### Study population

This study approved by CEIC Hospital Sant Pau included 202 consecutive patients with clinically suspected stable coronary artery disease (CAD) who underwent coronary computed tomographic angiography (CCTA). The study design, procedures, and population characteristics have been previously described [53, 54]. All participants were recruited from the Hospital de la Santa Creu i Sant Pau in Barcelona (Spain) and signed the informed consent for the study prior to their inclusion. Exclusion criteria included suspected or current acute coronary syndrome (ACS), severe infectious diseases, allergy to iodinated contrast agents, advanced renal failure (glomerular filtration rate < 30 mL/min/1.73 m^2^), body mass index > 40 kg/m^2^, inability to perform adequate breath-hold apnea, non-diagnostic CCTA due to artifacts, or the presence of any life-limiting condition.

Demographic, anthropometric, clinical, and pharmacological data were collected from electronic medical records at the time of the CCTA examination. High-sensitivity troponin T (hs-TnT) and C-reactive protein (hs-CRP) levels were measured using standardized procedures at the Biochemistry Service clinical laboratory of the Hospital de la Santa Creu i Sant Pau. Cardiac imaging was performed using CCTA, as detailed in the following section.

### Coronary computed tomograghy angiography (CCTA)

CCTA exams were performed using a 256-row CT system (iCT, Philips Healthcare, Amsterdam, The Netherlands) in the Cardiac Imaging Unit at the Hospital de la Santa Creu i Sant Pau, as previously described [53, 54]. The CT images were analyzed by two level-3 cardiac CCTA readers, who were blinded to the biomarker study. These readers interpreted the images and assigned patients to the appropriate study groups. The coronary arteries were evaluated using a 16-segment model, which is a modified 15-segment model that includes the intermediate branch as segment 16.

Luminal diameter stenosis was graded as follows: 0% (none), 1–29% (minimal), 30–49% (mild), 50–69% (moderate), 70–99% (severe), and 100% (total occlusion). The Segment Involvement Score (SIS) and Segment Stenosis Score (SSS) were calculated to assess the extent and severity of coronary atherosclerosis, serving as measures of coronary atherosclerotic burden. The SIS, which ranges from 0 to 16, was calculated by counting the total number of coronary artery segments exhibiting plaques, regardless of the degree of luminal stenosis. The SSS was used to evaluate the severity of coronary atherosclerosis and assigned points as follows: 0 points for stenosis of 0–29%, 1 point for 30–49%, 2 points for 50–69%, and 3 points for stenosis of 70% or greater. The individual scores from all 16 segments were summed to yield a total score ranging from 0 to 48. The prognostic value of these coronary artery plaque scores has been well-established in patients with suspected stable CAD, as demonstrated by Min et al. in 2007 and validated by independent groups [55–57]. According to the Coronary Artery Disease Reporting and Data System (CAD-RADS) 2.0 by Cury et al. [58] a cutoff was proposed to differentiate between mild to moderate CAD (SIS or SSS ≤ 4) and severe or extensive CAD (SIS or SSS > 4*).* Finally, a 3-vessel plaque score (no/yes) was also evaluated, considering the involvement of the left anterior descending, left circumflex, and right coronary arteries, regardless of the severity of the lesions.

### Culture of human coronary vascular smooth muscle cells

Human coronary vascular smooth muscle cells (VSMCs) from a single batch lot (ATCC-PCS-100-021, ATCC, lot #61 646 600) were used to minimize variability arising from different cell sources. To induce cell quiescence, cells were cultured for 24 h in medium containing 0.2% fetal bovine serum for 48 h in medium with 0.4% FCS at 37 °C and 5% CO2. For experiments, serum-starved cells between passages 4 and 8 were selected, as they formed a homogeneous population with a characteristic hill-and-valley pattern of confluence.

Cells were grown in vascular cell basal medium (ATCC-PCS-100-030) supplemented with components of the vascular smooth muscle growth kit (ATCC-PCS-100-042). Western blot analysis of differentiation markers confirmed high levels of α-actin (45 kDa) and calponin (33 kDa) in these cells. For culture, medium 199 was used, supplemented with 20% FBS, 2% human serum, 2 mmol/liter L-glutamine, 100 units/ml penicillin G, and 100 µg/ml streptomycin. Quiescent VSMCs were exposed to native or aggregated LDL for 2 h in proteomics studies and for 24 h in normoxic/hypoxic comparative studies. LDL aggregation was confirmed by turbidimetric analysis prior to cell incubation.

### LDL preparation and modification

Human LDLs (density 1.019–1.063 g/mL) were isolated from pooled serum of normocholesterolemic volunteers using ultracentrifugation. The LDLs were dialyzed overnight against 150 mmol/L NaCl, 1 mmol/L EDTA, and 20 mmol/L Tris–HCl (pH 7.4). Protein and cholesterol concentrations were determined using standard methods, with an average cholesterol content of ≈2 mg/mg LDL protein. LDL purity was confirmed by agarose gel electrophoresis. Aggregated LDLs were formed by vortexing LDLs in PBS and monitored by turbidimetry at 680 nm. The fraction of LDLs that aggregated (≥ 95%) after 4 min of vortexing was used for experiments, as previously described [5, 6].

### Exposure of hcSMC to LDL under normoxic and hypoxic conditions

SMCs were deprived of serum upon reaching 80% confluence. The cells were then unexposed or exposed to nLDL (control SMCs) or to aggregated LDL (foam-SMCs) under normoxia (21% O₂) in an incubator containing a gas mixture of 74% N₂ and 5% CO₂, or hypoxia (1% O₂) in a Hypoxic/Anoxic Workstation H35 (Don Whitley Scientific Ltd.) with a gas mixture of 94% N₂ and 5% CO₂, for 24 h as previously described [26, 27]. Following the treatment, cells were harvested by scraping them into NaOH for neutral lipid analysis or for Tripure^™^ Isolation Reagent (Roche Molecular Diagnostics) for subsequent PCR and Western blot analyses in SMC extracts.

### Thin layer chromatography of neutral lipid content in SMCs

The intracellular cholesteryl ester (CE), triglyceride (TG), and free cholesterol (FC) content in SMCs extracts was measured using thin-layer chromatography (TLC) after lipid extraction. Lipids were extracted with a dichloromethane/methanol (1:2) solvent mixture, and CE, TG and FC were separated on silica G-24 TLC plates as previously described [5, 6]. Lipid spots identified as CE, TG, and FC using standard curves for cholesterol palmitate, triglycerides, and cholesterol were quantified by densitometry and foam SMC formation was evaluated by the magnitude of intracellular CE/FC ratio induced by SMC exposure to LDL.

### Proteomic analysis

#### Immunoprecipitation of the SMC secretome

To compare the secretomes of control SMCs—both unexposed and exposed to native LDL (2 h)—and foam SMCs (exposed to aggregated LDL for 2 h), the total protein content of the corresponding supernatants was collected. Additionally, supernatants from SMCs exposed to aggregated LDL in the presence of an anti-LDL aggregation peptide (DP3) were also analyzed to establish specificity criteria for identifying proteins uniquely secreted by foam cells, as previously shown by our group [59, 60]. Total proteins from the supernatants were precipitated using trichloroacetic acid (TCA), collected by centrifugation, washed to remove residual contaminants, and resuspended in a suitable buffer for proteomic studies. Protein concentrations in the extracts were determined using the 2D-Quant Kit (GE Healthcare).

### Mass spectrometry analysis of differential proteomics in foam-SMC secretome

Proteins were identified after in-gel tryptic digestion and extraction of peptides from the gels pieces by matrix-assisted laser desorption/ionization time-of-flight using an AutoFlex III Smartbeam MALDI-ToF/ToF (Bruker Daltonics). Samples were applied to Prespotted AnchorChip plates (Bruker Daltonics) surrounding the calibrants provided on the plates. Spectra were acquired with flexControl on reflector mode (mass range 850–4000 m/z, reflector 1: 21.06 kV; reflector 2: 9.77 kV; ion source 1 voltage: 19 kV; ion source 2: 16.5 kV; detection gain 2.37x), with an average of 3500 added shots at a frequency of 200 Hz. Each sample was processed with flexAnalysis (Version 3.0, Bruker Daltonics) considering a signal-to-noise ratio over 3 and applying statistical calibration. For identification, peaks between 850 and 1000 were not considered, as in general only matrix peaks are visible on this mass range. After processing, spectra were sent to the interface BioTools (Bruker Daltonics, version 3.2) and, with no further modifications, MASCOT search on Swiss-Prot 57.15 database was done (Taxonomy: Homo Sapiens, Mass Tolerance 50–100, up to 2 miss cleavage, Global Modification: Carbamidomethyl [C], Variable Modification: Oxidation [M]). Identification was accepted with a score higher than 56. Inhibitors of foam cell formation [59, 60] were employed to confirm the specific foam-SMC origin of the selected differential proteins.

Results of the amount of protein in Mass Spectrometry Analysis of Differential Proteomics are expressed as a normalized abundance on a log2 scale.

The samples were processed in triplicate for each group, and peptides were identified through a database search with a false discovery rate (FDR) of 1%. To identify proteins specifically secreted by foam cells and differentially expressed upon exposure to DP3, we established the following specificity criteria: a fold change of ≥ 1 between the nLDL and agLDL conditions, a fold change of ≤ −1 between the agLDL and agLDL with DP3 peptide (agLDL_DP3) conditions, and a fold change between −0.3 and 0.3 between the agLDL and agLDL with the irrelevant peptide (agLDL_IP321) conditions. Using these criteria, we identified six proteins, including EPAC1.

### Reverse transcription and real-time qPCR analysis

Total RNA from SMCs was extracted using the Total RNA Extraction Kit (Sigma) and quantified with a NanoDrop ND-1000 spectrophotometer (NanoDrop Technologies). The RNA was reverse-transcribed into cDNA using the RevertAid First Strand cDNA Synthesis Kit (Thermo Scientific). The 18S rRNA gene served as the housekeeping control. Quantitative real-time PCR (qRT-PCR) was conducted using TaqMan assays for Exchange Protein Directly Activated by cAMP 1 (EPAC1) (Thermofisher, Hs01030417_m1) on a 7900HT Fast Real-Time PCR System (AB-Thermo Fisher Scientific). We mixed 5 μl of single-stranded cDNA (equivalent to 100 ng of total RNA) with 1 μl of 20 × TaqMan Gene Expression Assays for each Assay-on-Demand, 10 μl of TaqMan Universal PCR Master Mix, and 4 μl of nuclease-free water. After gentle mixing, the mixture was transferred into a real-time PCR microplate. The Real-time PCR microplate was sealed, centrifuged, and then was placed in the sample block of an Applied Biosystems 7300 Real Time PCR System (Applied Biosystems). The thermal cycling conditions were 2 min at 50 °C and 10 min at 95 °C, followed by 40 cycles of 15 s at 95 °C and 1 min at 60 °C. Expression levels were measured in triplicate. The threshold cycle (Ct) values were normalized to the housekeeping gene.

### Western blot analysis

Proteins were isolated using TriPure^™^ Isolation Reagent (Roche) according to the manufacturer’s instructions. The protein samples were then analyzed by Western blot as previously described [5, 6] Blots were incubated with monoclonal antibodies against EPAC1 (Santa Cruz, sc-28366). To ensure equal protein loading across samples, blots were stained with Ponceau, and a normalization of results for gapdh (Abcam, ab8245) was performed.

### Enzyme-linked ImmunoSorbent assay (ELISA) for detection of EPAC1

The concentration of EPAC1 was determined in human serum from patients using a quantitative sandwich ELISA kit obtained from MyBioSource (MBS7607909, Vizcaya, Spain), which has a sensitivity of 10 pg/mL.

### Classical protein-based biomarker determination

hs-TnT and hs-CRP as classical protein biomarkers were compared against the diagnostic performance of EPAC1. hs-TnT concentrations were measured by electro-chemiluminiscence immunoassay using the hs-TnT on the Roche Cobas e601 analyzer (Roche Diagnostics, Mannheim, Germany). The hs-TnT assay has an analytic range from 3 to 10000 ng/L and showed no significant cross-reactivity with TnT. The assays had inter-run coeficients of variation ranging from 1.2 to 3.7%. hs-CRP concentrations were determined using and immunoturbidimetry method on the Roche Cobas c501 analyzer (Roche Diagnostics). The hs-CRP assay has an analytic range from 0.3 to 350 mg/L. The assay had inter-run coeficients of variation ranging from 1.2 to 3.6%.

### Statistical analysis

EPAC1 levels were initially compared between patients categorized into CAD and non-CAD groups. Subsequently, EPAC1 levels were analyzed in groups stratified by bioimaging markers of CAD severity, including SIS (0, 1–4, > 4), SSS (0, 1–4, > 4), stenosis (0, < 50%, > 50%), and the 3v-score (no/yes). Descriptive statistics were used to summarize the study population's characteristics, and the Kolmogorov–Smirnov test was applied to assess normality. Continuous variables with normal distributions were presented as the mean ± standard deviation (SD), while those with skewed distributions were reported as the median ± interquartile range (IQR). Continuous variables were compared between groups using Student’s t test and Mann–Whitney U test for variables with skewed distributions. Categorical variables were expressed as frequencies (percentages) and compared using Fisher’s exact test. Spearman’s Rho coefficient was used to evaluate correlations between nonparametric variables, while Pearson’s correlation coefficient was applied for parametric variables. Logistic regression analyses were conducted to explore in detail the association between potential biomarkers and CAD indices. To determine whether the observed association could be influenced by potential confounding factors, three models were analyzed. In Model 1, conventional cardiovascular risk factors (age and dyslipidemia) were included as covariates. Model 2 incorporated additional factors, such as BMI, hypertension (HTA), diabetes mellitus (DM), and high-sensitivity troponin T (hs-TnT). Finally, Model 3 included sex as a covariate alongside age and dyslipidemia. Receiver operating characteristic (ROC) curves were generated for EPAC1, with the area under the ROC curve (AUC) used as a measure of overall discrimination. The Youden Index was employed to identify the optimal cutoff point [61]. The additional discriminatory capacity of EPAC1 beyond a clinical model based on conventional cardiovascular risk factors was also assessed. The results were reported as R^2^, standard error (SE), and p-value. The statistical software Package SPSS 29 for Windows (SPSS Inc. Chicago, IL, USA) was used for all statistical analyses. Differences were considered statistically significant when p ≤ 0.05.

## Results

### Foam-SMC secrete differential levels of EPAC1 into the extracellular medium

**Fig. 1:**
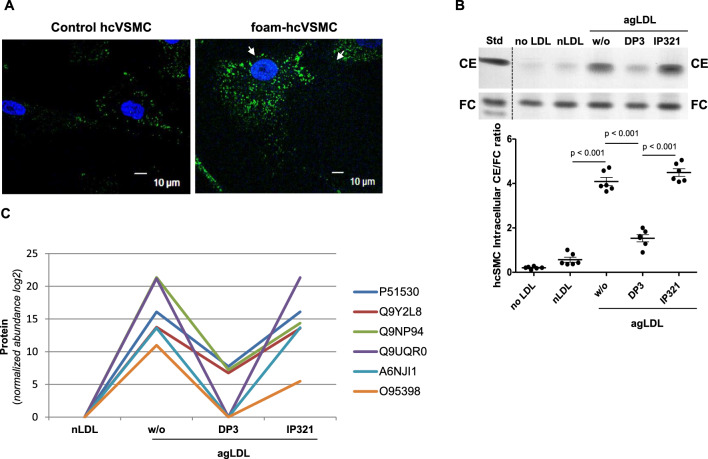
Differential mass spectrometry analysis comparing the secretoma of control and foam human coronary smooth muscle cells (hcVSMC). **A** Confocal microscopy images showing control (exposed to native LDL) and foam human coronary smooth muscle cells (hcSMC) generated through exposure to aggregated LDL (agLDL). White arrows indicate the abundance of lipid droplets (LDs) in foam-hcSMC compared to control hcSMC. Scale 10 µm. **B** Thin layer chromatography (TLC) analysis of intracelular cholesteryl ester (CE)/free cholesterol (FC) ratio (marker of foam cell formation) in hcSMCs exposed to aggregated LDL (agLDL) (foam SMC) in absence (w/o) or presence of a foam cell inhibitor peptide (DP3) or an irrelevant peptide (IP321) for 2 h. Control cells were those unexposed to LDL (no LDL) or exposed to native LDL (nLDL). Results are shown as mean ± SD of three experiments performed in duplicate. **C** Results from the differential mass spectrometry analysis of secretomes in the conditions of native LDL (nLDL), aggregated LDL (agLDL) without (w/o) peptides or with inhibitor peptide (DP3) or an irrelevant peptide (IP321). O955398 (EPAC1) was one of the six proteins that met the specificity criteria for foam-hcSMCs

Figure 1A illustrates the abundance of lipid droplets (white arrows) in hcVSMCs exposed to aggregated LDL (foam-hcVSMC) compared to those exposed to native LDL (hcVSMC). Thin-layer chromatography confirmed the hcVSMC transformation into foam cells upon exposure to aggregated LDL, as evidenced by a significant increase in intracellular CE/FC ratio compared to control hcSMCs (unexposed or exposed to native LDL) (Fig. 1B). To identify proteins specifically and differentially secreted by foam hcVSMCs, we conducted a differential mass spectrometry analysis of foam-hcVSMC and control hcVSMC secretomes (Fig. 1C). Three biological replicates were processed for each group, and peptides were identified through a database search with a 1% false discovery rate (FDR). Applying the specificity criteria described in Methods, EPAC1 (O95398, orange line) was identified as one of the differentially secreted proteins by foam SMCs.

### Blood EPAC1 levels are lower in individuals with CAD compared to those without CAD

A total of 202 patients were included. They were initially divided into a control group without CAD (n = 50; 15 men and 35 women) and a group with CAD (n = 152; 99 men and 53 women). Table 1 summarizes the main clinical and imaging characteristics of patients included in this study, which included individuals with clinically suspected stable CAD who underwent a CCTA exam.

**Table 1:** Clinical, metabolic, coronary artery bioimaging, and blood biomarker levels in the study population (Control and CAD groups), analyzed by total population, men, and women

	Total			Women (W)			Men (M)		
	Control (n = 50)	CAD (n = 152)	p	Control (n = 35)	CAD (n = 53)	p	Control (n = 15)	CAD (n = 99)	p
Clinical characteristics									
Sex, M(%)–W(%)	15(30)–35 (70)	99 (65.1)–53 (34.9)	< 0.001	–	–	–	–	–	–
Age (y), md (IQR)	54 (43–65)	69 (62–76)	< 0.001	55 (44–66)	73 (66–79)	< 0.001	52 (41–61)	68 (58–74)	< 0.001
BMI (W/H^2^), md (IQR)	25.05 (23.58–28.13)	27.76 (24.91–30.82)	0.001	24.51 (23.31–27.50)	27.30 (24.22–31.22)	0.017	25.82 (25.00–29.39)	27.76 (25.55–30.45)	0.145
HTA, n (%)	21 (42)	108 (71.1)	< 0.001	14 (40)	39 (73,6)	0.002	7 (46.7)	69 (69.7)	0.087
DLP, n (%)	16 (32)	97 (63.8)	< 0.001	14 (40)	30 (56.6)	0.191	2 (13,3)	67 (67.7)	< 0.001
DM, n (%)	4 (8)	40 (26.3)	0.006	4 (11.4)	16 (30,2)	0.041	0 (0)	24 (24.2)	0.033
Smoker, n (%)	10 (21.3)	58 (39.2)	0.020	8 (24.3)	9 (18)	0.521	2 (14.3)	49 (50)	0.010
Glomerular filtration rate < 60 ml/min/1.73 m^2^, n (%)	5 (10.2)	18 (12.6)	0.658	5 (14.7)	8 (16.3)	0.843	0 (0)	10 (10.6)	0.187
Medications									
Statins, n (%)	15 (30.6)	86 (60.1)	< 0.001	12 (35.3)	26 (53.1)	0.112	3 (20)	60 (63.8)	0.001
Angiotensin-converting-enzyme inhibitors, n (%)	17 (34.7)	89 (62.2)	< 0.001	10 (29.4)	31 (63.3)	0.003	7 (46.7)	58 (61.7)	0.273
Betablokers, n (%)	10 (20.4)	56 (39.4)	0.016	8 (23.5)	16 (32.7)	0.370	2 (13.3)	40 (43)	0.029
Antiagregants, n (%)	13 (27.1)	72 (50.7)	0.005	11 (33.3)	23 (46.9)	0.223	2 (13.3)	49 (52.7)	0.005
Sintrom, n (%)	3 (6.1)	21 (14.7)	0.119	1 (2.9)	6 (12.2)	0.136	2 (13.3)	15 (16)	0.796
Diuretics, n (%)	7 (14.3)	46 (32.2)	0.016	6 (17.6)	20 (40.8)	0.026	1 (6.7)	26 (27.7)	0.082
Biochemistry									
Glucose (mg/dl), md (IQR)	90.00 (84.00–96.50)	100.50 (90.00–122.00)	< 0.001	88.00 (84.00–96.50)	97.00 (87.75–122.25)	0.009	91.00 (84.00–97.00)	101.00 (90.00–121.50)	0.014
Creatinine (mg/dl), md (IQR)	0.79 (0.67–0.97)	0.85 (0.75–1.08)	0.023	0.76 (0.62–0.86)	0.75 (0.69–0.90)	0.426	0.96 (0.79–1.08)	0.94 (0.79–1.11)	0.583
Urea (mg/dl), md (IQR)	5.40 (4.50–6.70)	6.20 (5.10–7.40)	0.023	5.15 (3.95–6.60)	6.20 (4.90–7.95)	0.113	6.10 (4.80–7.05)	6.15 (5.17–7.32)	0.687
Lipid profile									
Total Cholesterol (mg/dl), mean ± SD	202.92 ± 42.51	185.40 ± 40.96	0.013	205.94 ± 41.60	186.29 ± 43.83	0.045	195.57 ± 45.38	184.90 ± 39.53	0.353
LDLc (mg/dl), md (IQR)	117.00 (82.00–154.50)	99.00 (80.00–133.00)	0.094	125.00 (85.50–153.00)	97.50 (76.50–138.25)	0.126	91.00 (70.00–161.00)	100.00 (85.25–130.00)	0.860
HDLc (mg/dl), md (IQR)	60.50 (45.50–69.00)	49.00 (42.00–58.00)	0.004	63.00 (51.50–69.50)	53.00 (45.00–59.00)	0.005	44.00 (36.00–64.00)	48.00 (41.75–57.00)	0.885
VLDLc (mg/dl), md (IQR)	16.50 (11.25–21.75)	19.00 (16.00–27.00)	0.093	16.50 (15.00–20.50)	20.00 (15.75–28.25)	0.129	16.00 (10.00–22.75)	19.00 (16.00–24.00)	0.456
Triglycerides (mg/dl), md (IQR)	96.00 (69.00–121.00)	111.00 (88.50–151.00)	0.003	94.00 (68.00–119.50	114.50 (81.50–152.50)	0.027	97.00 (70.50–138.25)	110.00 (91.25–151.00)	0.121
CAD bioimaging									
Maximum stenosis, md (IQR)	0.00 (0.00–0.00)	2.00 (1.00–3.00)	< 0.001	0.00 (0.00–0.00)	2.00 (1.00–2.00)	< 0.001	0.00 (0.00–0.00)	2.00 (1.00–4.00)	< 0.001
3 vessel plaque score, n (%)	0 (0)	71 (46.7)	< 0.001	0 (0)	19 (35)	< 0.001	0 (0)	52 (52.5)	< 0.001
SIS, md (IQR)	0.00 (0.00–0.00)	5.00 (3.00–8.00)	< 0.001	0.00 (0.00–0.00)	3.00 (2.00–6.00)	< 0.001	0.00 (0.00–0.00)	6.00 (3.00–8.00)	< 0.001
SSS, md (IQR)	0.00 (0.00–0.00)	3.00 (1.00–7.75)	< 0.001	0.00 (0.00–0.00)	2.00 (1.00–4.00)	< 0.001	0.00 (0.00–0.00)	4.00 (1.00–9.00)	< 0.001
Blood biomarkers									
hs-TnT (ng/dl), md (IQR)	6.89 (4.46–10.65)	14.60 (7.99–21.24)	< 0.001	6.89 (4.03–11.85)	10.20 (6.06–18.89)	0.032	5.51 (4.86–9.26)	15.60 (8.66–22.19)	< 0.001
hs-CRP (mg/dl), md (IQR)	1.25 (0.65–2.55)	2.21 (1.09–5.17)	0.010	1.20 (0.76–3.11)	2.52 (1.51–5.61)	0.016	1.42 (0.49–2.10)	2.10 (0.99–4.92)	0.084
EPAC1 (ng/ml), mean ± SD	11.97 ± 3.30	9.41 ± 4.00	< 0.001	11.18 ± 3.20	9.45 ± 3.46	0.062	14.10 ± 2.68	9.40 ± 4.22	0.002

Results are expresed as median (md) with interquartile range (IQR) for non-parametric variables or mean with Standard Desviation (SD) for parametric variables. *BMI* Body mass index (kg/m^2^), *HTA* Arterial hypertension, *DLP* dyslipemia, *DM* Diabetes mellitus type 2, *LDLc* Low density lipoprotein cholesterol, *HDLc* High density lipoprotein cholesterol, *VLDLc* Very low density liporotein colesterol, *SIS* Segment involvement score, *SSS* Segment stenosis score, *hs-TnT* High sensitivity Troponin T, *hs-CRP* High sensitivity C reactive protein. T-student test was used for parametric distribution variables. U-mann whitney test was used for non-parametric distribution variables

There is a clear and significant age difference between patients with CAD and the control group, observed both in the overall cohort and within the subgroups of men and women. The prevalence of elevated BMI was higher in patients with CAD, both in the overall group and specifically among women. The prevalence of dyslipidemia and smoking did not differ significantly between women with CAD and the control group. However, hypertension showed significant differences between CAD patients and controls in the overall group, as well as in women subgroup. The use of statins, ACE inhibitors, beta-blockers, antiaggregants, and diuretics was also more prevalent in CAD patients, in the total grup, and in both men and women subgroups.

Circulating EPAC1 concentrations were significantly lower in the CAD group compared to the non-CAD group (9.41 ± 4.00 vs 11.97 ± 3.30 ng/mL, p < 0.001). When separated by sex, the differences were significant in men (9.40 ± 4.22 vs 14.10 ± 2.68 ng/mL, p = 0.002) but do not reach significance in women (9.45 ± 3.46 vs 11.18 ± 3.20 ng/mL, p = 0.062) (Table 1). hs-TnT levels were significantly higher in CAD patients compared to controls for the total group, as well as for both men and women. Similarly, hs-CRP levels were significantly elevated in CAD patients compared to the control group when considering the total population and women, but this difference did not reach significance in men (Table 1).

It is interesting to note that, in individuals without detectable CAD, blood EPAC1 levels were significantly lower in women than in men (11.18 ± 3.20 versus 14.10 ± 2.68 ng/mL, p = 0.021) (Table 1). These results suggest the potential regulation of EPAC1 levels by hormonal and sex-specific cardiovascular risk factors.

### Blood EPAC1 levels decrease further in CAD patients with greater severity as assessed by CCTA bioimaging

**Fig. 2:**
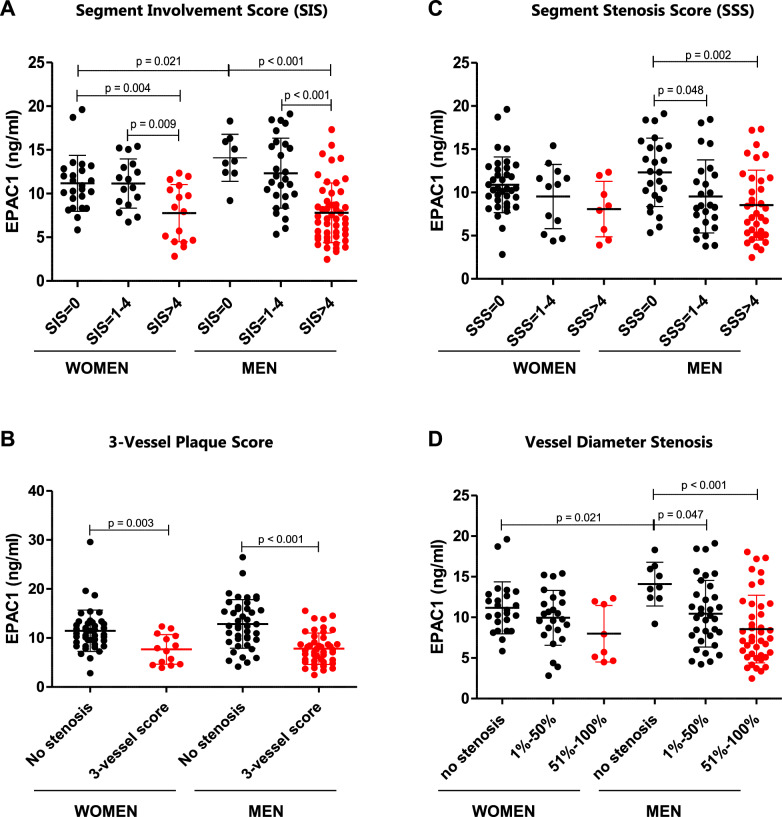
Comparison of blood EPAC1 levels across the absence, moderate, and severe stages of CAD in both women and men. Patients were grouped according to scores from bioimaging variables that determine the extent and severity of CAD. Blood EPAC1 levels were measured in patient groups segmented based on CAD severity as assessed by CCTA bioimaging variables, including the Segment Involvement Score (SIS) (**A**), 3-Vessel Plaque Score (**B**), Segment Stenosis Score (SSS) (**C**), and Vessel Diameter Stenosis (**D**). Comparisons between groups were conducted using a student’s t-test in parametric variables and mann–whitney U test for non-parametric variables, and results are presented as mean ± SD. Differences were considered statistically significant at p < 0.05. CAD, coronary artery disease, CCTA, coronary computed tomography angiography

Plasma levels of EPAC1 were significantly lower in patients with SIS > 4 compared to controls (SIS = 0) or moderate CAD (SIS 1–4) group, both in women (7.76 ± 3.27 vs 11.18 ± 3.20 ng/mL or 11.15 ± 2.82 ng/mL, p = 0.004 or p = 0.009, respectively) and men (7.80 ± 3.42 vs 14.10 ± 2.68 ng/mL or 12.32 ± 4.01 ng/mL, p < 0.001 for both comparisons) (Fig. 2A). However, no significant differences in EPAC1 levels were observed between the control and moderate CAD, in either women or men subgroups. These results suggest that EPAC1 has predictive value for severe CAD, but not for moderate CAD in relation to the SIS variable, regardless of gender.

We also analyzed EPAC1 protein levels by segmenting the population according to the 3-vessel score (Fig. 2B). EPAC1 levels were significantly lower in patients with 3-vessel score group compared to controls, either in women (p = 0.003) or men (p < 0.001). These results suggest that EPAC1 may be useful for detecting advanced stages of CAD in women and men.

Regarding SSS, significant differences in EPAC1 protein levels according to SSS were observed only in men. Specifically, EPAC1 levels decreased significantly from SSS = 0 (12.33 ± 3.95 ng/mL) to SSS = 1–4 (9.53 ± 4.22 ng/mL, p = 0.048) and further to SSS > 4 (8.54 ± 4.04 ng/mL, p = 0.002) (Fig. 2C). These results that EPAC1 has predictive value for moderate and severe CAD in men and in relation with SSS. In contrast, there were no significant differences according to SSS in women. Similarly, the analysis of maximal vessel stenosis in the epicardial coronary artery tree in men showed a significant decrease in EPAC1 protein levels, from 14.10 ± 2.68 ng/mL at 0% of stenosis to 10.46 ± 4.10 ng/mL in the < 50% stenosis (p = 0.047), and further to 8.55 ± 4.17 ng/mL in the ≥ 50% stenosis group (p < 0.001) (Fig. 2D). These results suggest that EPAC1 is a useful predictor of moderate to severe CAD in men, particularly concerning stenosis bioimaging variables in this cohort.

The differences in the potential diagnostic capacity of EPAC1 in CAD between men and women observed in this cohort should be interpreted with caution, as these results may be at least partly due to the limited number of participating women.

### Variation in current biomarkers for moderate and severe CAD between men and women

Differently to EPAC1, there were no differences in the levels of the current blood biomarkers hs-CRP (*online* Figure S1) or hs-TnT (*online* Figure S2) by gender in individuals without CAD for any of the bioimaging variables analyzed.

In women, there was a significant increase in hs-CRP levels in the SIS 1–4 group (p = 0.016) (*online* Figure S1 A), the SSS 1–4 group (p = 0.034) (*online* Figure S1 C), and the stenosis 1–50% group (p = 0.032) (*online* Figure S1D) compared to the respective control groups. However, no significant differences in hs-CRP levels were observed based on 3-vessel plaque score in this gender (*online* Figure S1B). In men, hs-CRP only exhibited significant differences according to SIS > 4 *vs* SIS = 0 (p = 0.05) (*online* Figure S1 A) and according to the 3-vessel plaque score (p = 0.025) (*online* Figure S1B). These results suggest that hs-CRP has diagnostic utility for moderate CAD in women and for severe CAD in men, at least in this cohort.

Unlike hs-CRP, hs-TnT levels did not show significant differences in women segregated in groups according to CAD severity assessed by imaging biomarkers. In men, however, hs-TnT levels were significantly elevated in both the SIS 1–4 and SIS > 4 groups compared to controls (p = 0.016 and p < 0.001, respectively) (*online* Figure S2 A)., Additionally, hs-TnT levels were significantly higher in 3-vessel score and SSS > 4 groups compared to control groups (p < 0.001 and p = 0.015, respectively) (online Figure S2B, S2 C). hs-TnT levels also rose significantly from the control group to the stenosis < 50% group (p = 0.005), with an even greater increase in the stenosis ≥ 50% group (p < 0.001) (*online* Figure S2D). These results validate the potential of hs-TnT as a predictor of CAD severity in men.

### EPAC1, but not the classical biomarkers, correlates with imaging variables of CAD severity in patients

**Fig. 3:**
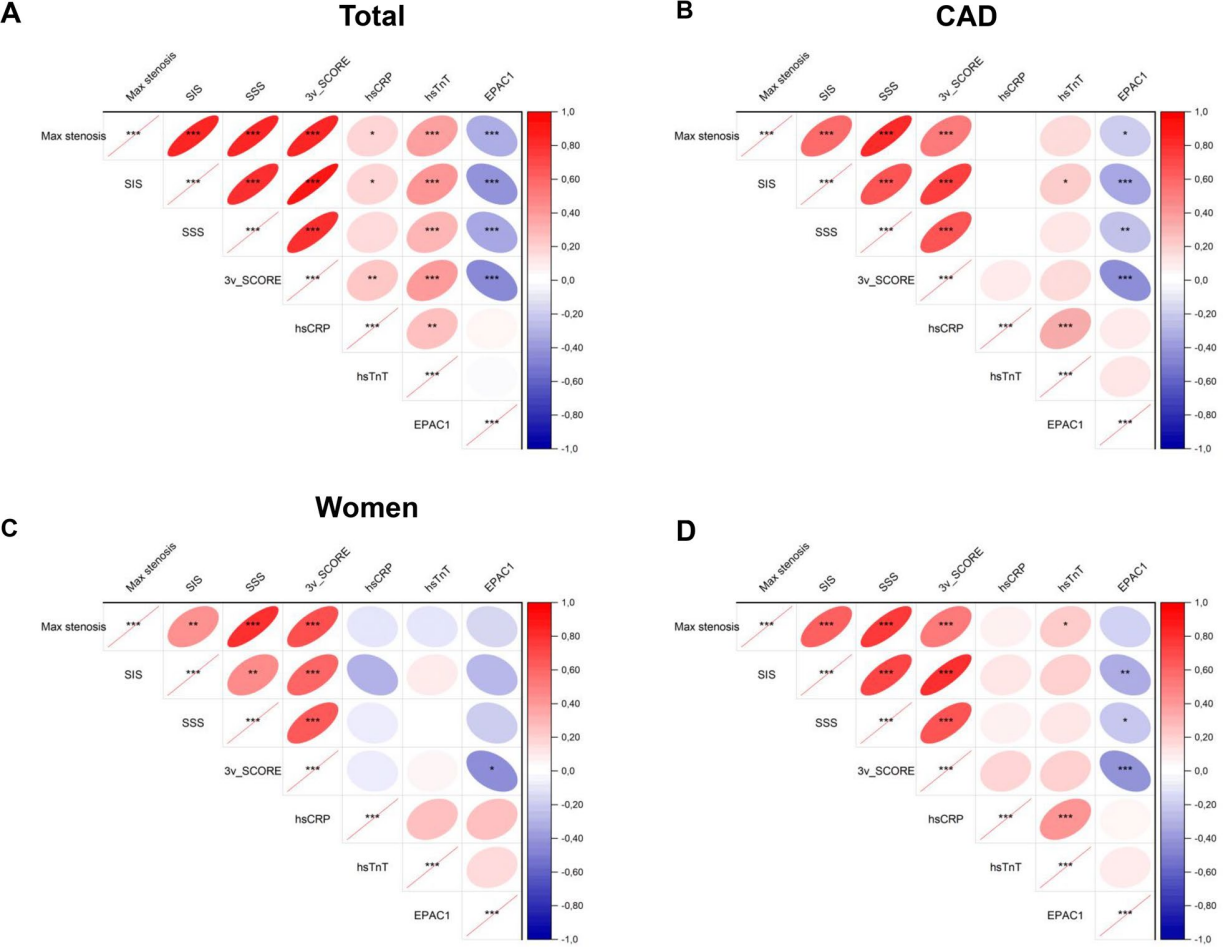
Correlograms showing correlations between CCTA bioimaging variables and blood protein biomarkers. Spearman’s correlations between CCTA bioimaging variables and blood protein biomarkers in the total subjects (**A**), CAD subjects (**B**), CAD Women (**C**) and CAD Men (**D**). Positive correlations are shown in red and negative correlations in blue, with the intensity of the color representing the Spearman’s correlation coefficient. The shape of each cell corresponds to the confidence ellipse of the scatter plot between variables. Significant levels are indicated with asterisks. *p ≤ 0.05 **p ≤ 0.01 ***p ≤ 0.001. CAD, coronary artery disease, CCTA, coronary computed tomography angiography

Correlograms illustrate the inverse or direct correlations of CAD bioimaging indices with circulating levels of EPAC1 or current biomarkers, respectively (Fig. 3 and *online* Table S1). In the overall population (Fig. 3A), EPAC1 exhibited a similar level of association with SIS, SSS, stenosis and the 3-vessel score as hs-TnT (p ≤ 0.001 for all). In contrast, hs-CRP displayed weaker associations with stenosis, SIS and the 3-vessel score compared to hs-TnT and EPAC1. In CAD patients (Fig. 3B), hsCRP did not show significant correlations with CAD bioimaging parameters, while hs-TnT exhibited only a weak correlation with SIS (p = 0.018). In contrast, EPAC1 showed notable correlations with SIS (p < 0.001) and the 3-vessel score (p < 0.001), alongside lower correlations with stenosis (p = 0.033) and SSS (p = 0.008). In women, neither hs-CRP or hs-TnT showed significant correlations with any CAD imaging variables, while EPAC1 exhibited a correlation with 3-vessel plaque score (p < 0.05) (Fig. 3C). In men, hs-TnT showed a weak correlation with stenosis (p = 0.05), whereas EPAC1 had strong correlations with SIS and the 3-vessel score (p < 0.01 for SIS and p < 0.001 for 3-vessel score) (Fig. 3D). Collectively, these findings suggest that EPAC1 exhibits a stronger correlation with CCTA imaging indices of CAD compared to hs-TnT and hs-CRP, both in the overall CAD population and within male and female subgroups.

### Independent association of EPAC1 with CAD severity

Table 2 summarizes the results of multiple linear regression models evaluating the impact of EPAC1 and other key variables on SIS. Model 1, using the enter method, achieved an R^2^ of 0.349, indicating that 34.9% of the variance in SIS was explained by age, EPAC1, and dyslipidemia. Model 2, which included additional variables, showed that EPAC1 remained independently associated with SIS, achieving an improved R^2^ of 0.366. Finally, Model 3, which was simplified to include only four variables, explained 46.2% of the variance in SIS, with EPAC1 consistently demonstrating an independent association. In addition, an ANCOVA analysis was conducted to assess the significance of sex in the relationship between SIS and EPAC1. When the sex variable was included as a covariate in a univariate analysis of variance, the relationship remained significant. Notably, the SIS F-value (F = 5.311; p < 0.001) was higher than the F-value for sex (F = 2.369; p = 0.126) (online Table S2).

**Table 2:** Multivariate Analysis to identify predictors of SIS

Variable	F_(3,140)_	R^2^	B	SE	P
Model 1 (ENTER METHOD)	25.002	0.349	−0.639	1.759	0.718
Age			0.095	0.023	< 0.001
EPAC1			−0.203	0.059	< 0.001
DLP			1.456	0.543	0.008

Variable	F_(7,129)_	R^2^	B	SE	P
Model 2 (ENTER METHOD)	10.653	0.366	−1.039	2.418	0.668
Age			0.071	0.027	0.010
BMI			0.064	0.059	0.280
HTA			0.632	0.620	0.310
DLP			1.444	0.586	0.015
DM			−0.322	0.706	0.649
Hs-TnT			0.007	0.009	0.452
EPAC1			−0.233	0.064	< 0.001

Variable	F_(4,139)_	R^2^	B	SE	P
Model 3 (ENTER METHOD)	29.872	0.462	−1.833	1.620	0.260
EPAC1			−0.201	0.053	< 0.001
Sex			2.482	0.458	< 0.001
Age			0.091	0.021	< 0.001
DLP			1.307	0.496	0.009

A large F-statistic value indicates that the regression model effectively explains the variation in the dependent variable. The R^2^ value represents the percentage of variation in the dependent variable (SIS) explained by the model. The β-coefficient (B) shows how much the dependent variable (SIS) changes with each unit change in the independent variable. The standard error (SE) reflects the potential error associated with the β-coefficient (B). *DLP* dyslipidemia, *BMI* body mass index (kg/m^2^), *HTA* arterial hypertension, *DM* type 2 diabetes mellitus, *hs-TnT* high-sensitivity troponin T

### EPAC1 as a potential biomarker of CAD severity

**Fig. 4:**
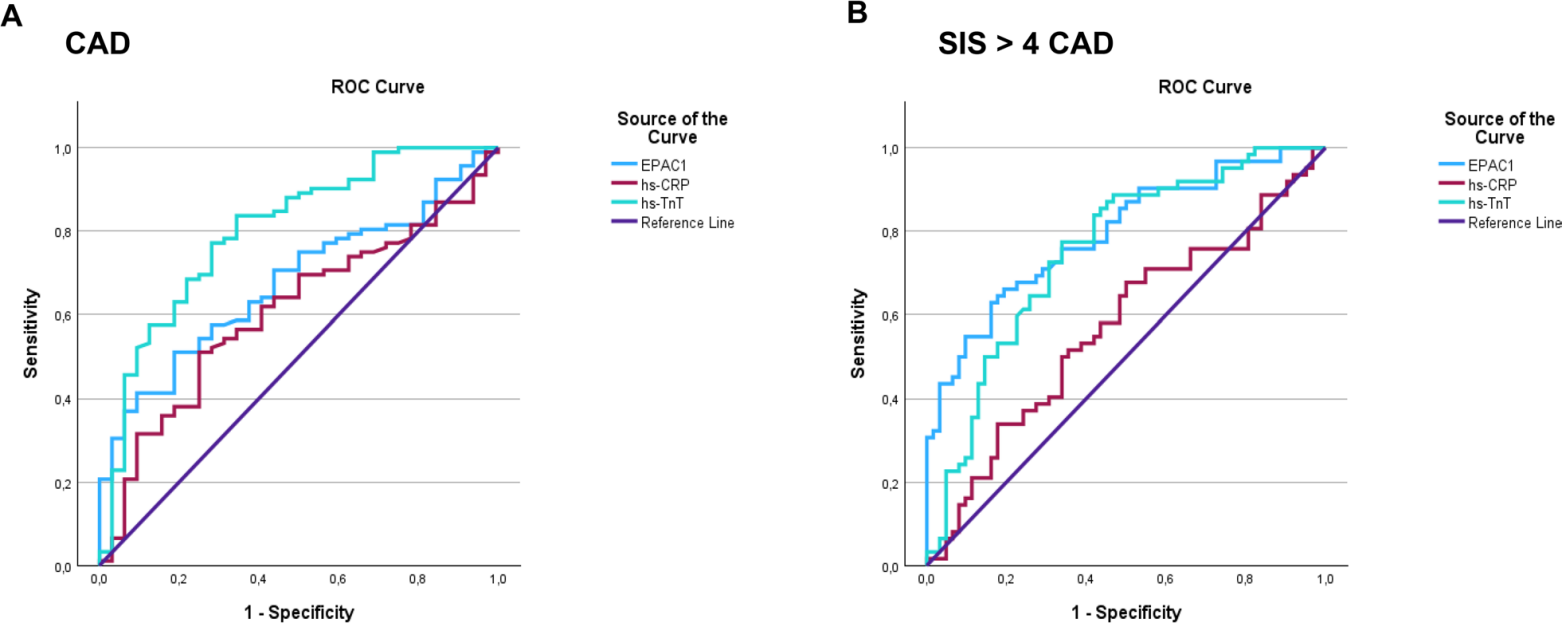
ROC curve analyses comparing the predictive capacity for CAD of EPAC1, hs-CRP, and hs-TnT. The predictive capacities for CAD (**A**) and severe CAD (**B**) are shown. CAD, coronary artery disease

A receiver operating characteristic (ROC) analysis was performed to evaluate the sensitivity and specificity of EPAC1 levels in predicting CAD presence (Fig. 4A) and CAD severity (SIS > 4) (Fig. 4B). The youden´s index analysis [37] determined that the optimal EPAC1 cut-off point was 9.16 ng/ml for both curves. At these cut-off value, EPAC1 predicted the probability of CAD presence with a sensitivity of 51.1% and a specificity of 81.2% (Table 3), and predicted the probability of severe CAD (SIS > 4) with a sensitivity of 69.6% and a specificity of 79.4% (Table 4). The performance of EPAC1 was superior to that of hs-CRP and hs-TnT in predicting CAD severity measured by SIS > 4 (AUC for EPAC1 = 0.814 *vs* AUC for hs-TnT = 0.723 and AUC for hs-CRP = 0.576) (Table 4). However, it was not as effective as hs-TnT in predicting the presence of CAD (AUC for EPAC1 = 0.677 *vs* AUC for hs-TnT = 0.801) (Table 3).

**Table 3:** ROC curve 1. CAD is the state variable

Variable	AUC	95% CI	P
EPAC1	0.677	0.580–0.775	< 0.001
hs-CRP	0.606	0.499–0.714	0.051
hs-TnT	0.801	0.709–0.892	< 0.001

Results are presented as AUC (Area Under the Curve), 95% CI (Confidence Interval), and p-value. *hs-TnT* high-sensitivity troponin T, *hs-CRP* high-sensitivity C-reactive protein

**Table 4:** ROC curve 2. SIS > 4 is the state variable

Variable	AUC	95% CI	P
EPAC1	0.814	0.740–0.889	< 0.001
hs-CRP	0.576	0.474–0.678	0.142
hs-TnT	0.723	0.634–0.812	< 0.001

Results are expressed as AUC (Area Under the Curve), 95% CI (Confidence Interval), and p-value. *hs-TnT* high-sensitivity troponin T, *hs-CRP* high-sensitivity C-reactive protein

### EPAC1 levels dramatically decreased in foam-SMCs under hypoxia

**Fig. 5:**
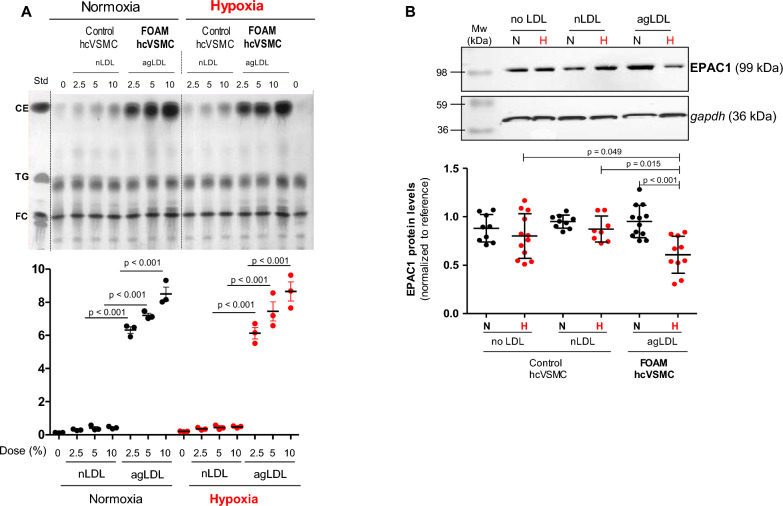
EPAC1 protein levels dramatically decreased in foam-hcVSMCs under hypoxia. **A** Thin-layer chromatography analysis of intracellular cholesteryl ester (CE), TG (triglyceride) and free cholesterol (FC) content of control hcVSMC (unexposed to LDL or exposed to native (nLDL)) or foam hcVSMC (exposed to aggregated LDL (agLDL) for 24 h). Bar graphs showing intracellular CE/FC ratio as marker of foam cell formation. Results are presented as the mean ± SD from three different experiments performed in duplicate **B** Western blot analysis of EPAC1 protein levels (n = 5 exp. by duplicate in normoxic conditions and n = 6 exp. by duplicate in hypoxic conditions). EPAC1 protein levels were normalized to normalized to the endogenous control gapdh and to reference values (SMC unexposed to LDL under normoxia). *hcVSMC* human coronary vascular smooth muscle cells, *N* normoxia, *H* hypoxia

Thin layer chromatography (TLC) showed the high potency of aggregated LDL to generate foam cell formation from human coronary VSMC either in normoxic or hypoxic conditions. Foam hcVSMCs exhibited a ninefold increase in the intracellular CE/FC ratio, far higher than in control cells exposed to native LDL, where the ratio remained nearly unchanged compared to cells unexposed to LDL (Fig. 5A). Real time PCR (*online* Figure S3) and Western blot (Fig. 5B) analysis, showed that there were no significant differences in EPAC1 levels (at mRNA and protein levels) between control and foam hcSMC under normoxic conditions. However, EPAC1 was strongly reduced in hypoxic foam-hcVSMC compared to normoxic foam-hcVSMC at both mRNA (p = 0.013) (*online* Figure S3) and protein (p < 0.001) (Fig. 5B) levels. EPAC1 levels were also reduced in hypoxic foam-SMC compared to hypoxic control SMC (unexposed or exposed to nLDL) both at mRNA (p < 0.001 vs both conditions) (*online* Figure S3) and protein (p = 0.049 and p = 0.015) (Fig. 5D) levels.

## Discussion

This study provides the first evidence supporting the clinical use of EPAC1 as a diagnostic biomarker for advanced CAD in both men and women with suspected stable CAD. Additionally, it sheds light on alterations in EPAC1 production and secretion by human coronary VSMCs (hcVSMCs), particularly foam-hcVSMC under hypoxic conditions. These findings suggest a potential association between decreased EPAC1 production by hypoxic foam-hcVSMCs, reduced blood EPAC1 levels, and the presence of advanced CAD in patients with suspected CAD. Addressing gender differences in the diagnostic potential of biomarkers is a key research priority. Therefore, we conducted a comprehensive analysis of all results from a gender-sensitive perspective. Our findings highlight the potential of EPAC1 as a unique and valuable biomarker for assessing CAD severity in both women and men. Unlike classical biomarkers, EPAC1 demonstrates a direct association with imaging-derived severity measures, suggesting its potential role in improving diagnostic accuracy and patient stratification.

Our key findings are as follows: (i) patients with advanced CAD in terms of extension and severity show decreased EPAC1 levels in blood; (ii) circulating EPAC1 is inversely associated with CAD imaging indices of disease extent and severity, even after adjusting for conventional cardiovascular risk factors; (iii) in women, where hs-CRP and hs-TnT show no significant correlation with CAD imaging measures, EPAC1 displays a strong inverse correlation with the SSS; (iv) EPAC1 has a lower discriminatory capacity than hs-TnT for CAD presence; (v) EPAC1 outperforms hs-CRP and hs-TnT in predicting CAD severity; and (vi) hypoxic coronary foam-hcVSMC may contribute to reduced EPAC1 levels in patients with advanced CAD.

Patients included in this study have been diagnosed according to the 2019 and 2024 European Society of Cardiology (ESC) guidelines for management of chronic coronary syndromes (CCS) [62, 63]. These guidelines recommend CCTA as the initial test for symptomatic patients with an intermediate-to-low probability of CAD. For patients with prior revascularization or established CCS, functional tests such as stress echocardiography or cardiac MRI are preferred to assess ischemia and clinical progression. Consequently, in our cohort, the number of patients with a history of myocardial infarction or revascularized CCS is minimal, making it unlikely to impact the overall results. By adhering to routine clinical practices and current ESC guidelines, we believe our study design accurately reflects real-world clinical care as implemented in most centers.

The results of the present study show that, in individuals without detectable CAD, blood EPAC1 levels are significantly lower in women than in men. Since reduced circulating EPAC1 levels are associated with a pathological state in our cohort, it is interesting to note, as highlighted in a recent review, that women face higher cardiovascular risk due to a greater incidence of classic risk factors such as diabetes, hypertension, obesity, and smoking. Additionally, women have specific risk factors (e.g., menarche, menopause) and conditions (e.g., autoimmune diseases, migraines) that could contribute to lower EPAC1 levels [64]. Further studies are needed to explore how these factors influence EPAC1 protein levels in both men and women and their implications for CAD severity. Both estrogens (E2) and testosterone have been shown to reduce EPAC1 levels in human airway smooth muscle cells, influencing Brain-Derived Neurotrophic Factor (BDNF), which plays a key role in inflammation, remodeling, and hyperreactivity [65].

The decision to analyze EPAC1 rather than EPAC2 in the blood of patients from this cohort was based on our proteomic study in human coronary smooth muscle cells. Among all the differentially expressed proteins identified, only five met the specificity criteria, and EPAC1—unlike EPAC2—was one of them. This finding aligns with existing knowledge, as EPAC1, but not EPAC2, is expressed in the vasculature and heart [32, 66], making its presence in human coronary SMCs biologically plausible. EPAC1, is mainly involved in regulating endothelial permeability and vascular tone, as well as cell migration and adhesion while EPAC2 is more more closely linked to metabolic diseases such as diabetes due to its role in insulin secretion and glucose homeostasis [39, 40].

We have evaluated EPAC1 in total plasma and not in isolated microvesicles because in terms of clinical applicability, it is preferable to avoid additional steps unless they are strictly necessary. There are various types of microvesicles secreted by SMC, and it could be valuable from the mechanistic point of view to investigate which of them carry EPAC1. However, for the purposes of this study, we believe that determining the total levels of EPAC1 in blood is a more clinically suitable approach. In future studies exploring the mechanisms involved in the secretion of this protein in other matrices, such as cell supernatants, it would be interesting to isolate and characterize EPAC1-positive microvesicles.

Currently, only one manuscript—besides ours—has documented the presence of EPAC1 protein in human blood. This study reported an upregulation of circulating EPAC1 in patients with type 2 diabetes treated with Glucagon-like Peptide-1 (GLP-1) receptor agonists (GLP-1RAs) [67]. The increase in EPAC1 in GLP-1Ras-treated diabetic patients was associated with significant reductions in HbA1c, glucose levels, LDL-C, body mass index (BMI), waist circumference, and diastolic blood pressure. According to the regression models used in our study, the association between EPAC1 and stenosis was independent of BMI, hypertension, dyslipidemia, and type 2 diabetes mellitus in our cohort.

This study found that hs-TnT is significantly associated with various CAD imaging variables, such as stenosis diameter and vessel scores, supporting previous research linking plaque burden to hs-TnT levels [48, 68–70]. In contrast, hs-CRP showed only a weak association with stenosis and vessel scores, consistent with studies indicating it rises in patients with advanced plaques [52]. Among CAD patients, hs-TnT had a weak association with SIS, significant only in men. Overall, our findings align with previous studies suggesting that hs-TnT and hs-CRP have limited value in predicting CAD burden, especially in stable CAD patients [52]. In this context, EPAC1 emerges as a blood protein biomarker that correlated with most CAD severity parameters across the entire cohort included in the current study, independently of baseline risk factors. This association with CAD bioimaging variables remained consistent when considering all CAD patients and, particularly in men. In women, only a strong correlation of EPAC1 with SSS was observed. It is important to note that the limited number of bioimaging variables associated with EPAC1 in women may stem from the smaller number of women with advanced CAD found in our cohort. This suggests that validating these results in a cohort with a more balanced representation of men and women would be advisable, and likely reinforce the diagnostic value of EPAC1 for CAD severity also in women. The progression of CAD from stable to unstable stages and acute events exhibits gender-specific differences in epidemiology, pathophysiology, and clinical presentation between women and men [71]. In younger age groups, the incidence of acute CAD-related events is lower in women than in men, but this trend reverses after 80 years of age. Although further studies of validation are indeed required, our study provided gender differences in blood EPAC1 levels and its diagnostic performance in CAD allowing to integrate a gender perspective into the future clinical application of this innovative biomarker.

A critical question addressed in this study is why EPAC1 levels are reduced in the blood of patients with advanced CAD. Insights into the underlying mechanisms can be inferred from experiments conducted here on cultured human coronary SMCs. Our results evidence that foam-hcVSMCs exposed to hypoxia suffer a significantly decrease in EPAC1 expression at both the mRNA and protein levels. These findings suggest that the strong predictive value of EPAC1 in identifying advanced plaque burden in patients may stem from the profound impact of hypoxia—a key driver of atherosclerosis progression [23–25]—on EPAC1 production by foam-hcVSMCs. Notably, foam-SMCs account for over 50% of foam cells in the arterial intima during intermediate stages of the disease [8–10].

The transcriptional regulation of EPAC1 remains poorly understood. It is known that EPAC1 transcription is inhibited by high cAMP levels [72] and upregulated by HIF-1α, a key hypoxia mediator, in CD34 + hematopoietic stem cells [29]. The results of the present study showed that hypoxia upregulated EPAC1 mRNA in control hcSMCs (although this effect did not reach statistical significance), while it downregulated EPAC1 mRNA in foam hcSMCs. This suggests that the impact of hypoxia on EPAC1 mRNA may vary depending on SMC phenotype, and that different SMC phenotypes in the plaque may have varying, and potentially opposing, effects on the progression of aterosclerosis.

EPAC1 has been reported to upregulate lectin-like oxidized low-density-1 (LOX-1) promoting foam cell formation from macrophages and atherosclerosis in a murine model [35]. LOX-1 appears to be expressed in SMCs, where its activation by oxLDL contributes to critical processes in atherosclerotic plaque development, including oxidative stress, inflammation, and mechanical stress [73–75]. It is highly relevant to investigate whether LOX-1 is modulated by EPAC1 in hcSMCs and to determine the extent to which this regulation depends on the hcSMC phenotype. Further studies are needed to compare LOX-1 levels in control and foam hcSMCs under both normoxic and hypoxic conditions. The hypoxia-driven mechanism described in this study that occurrs in a pathological SMC phenotype prevalent in advanced atherosclerotic plaques [8–10], may explain why EPAC1 outperforms current biomarkers in diagnosing severe CAD.

The key conclusions of this study are: (1) EPAC1, the primary EPAC isoform in vessels, is detectable in the blood of patients with suspected CAD; (2) EPAC1 demonstrates superior diagnostic performance compared to current biomarkers for identifying severe CAD in this population; and (3) hypoxia-driven mechanisms that suppress EPAC1 production in human coronary foam-SMCs—a major pathological SMC phenotype in atherosclerotic plaques—likely contribute to the reduced circulating EPAC1 levels observed in patients with advanced CAD.

The main limitations of this study are: the relatively small representation of women with advanced or severe CAD compared to men, and the absence of detailed information about the exact cellular sources of EPAC1 in the bloodstream.

## Supplementary Information

Supplementary material 1

Supplementary material 2

## Data Availability

The datasets used and/or analyzed during the current study are available from the corresponding author on reasonable request.

## Abbreviations

ACS: Acute coronary syndrome
BDNF: Brain derived neurotrophic factor
CAD: Coronary artery disease
cAMP: Cyclic adenosine MonoPhosphate
CCTA: Coronary computed tomographic angiography
CCS: Chronic coronary syndromes
CE: Cholesteryl esters
ELISA: Enzyme-linked ImmunoSorbent assay
EPAC: Exchange protein directly activated by cAMP
ESC: European Society of Cardiology
eQTL: Expression quantitative trait locus
FC: Free cholesterol
GEF: Guanine nucleotide exchange factor
GWAS: Genome-Wide Association Studies
hcVSMC: Human coronary vascular smooth muscle cells
hs-CRP: Highly sensitive C-reactive protein
hs-TnT: Highly-sensitivity troponin T
LDL: Low-density lipoproteins
LOX-1: Lectin-like OXidized low-density lipoprotein receptor-1
LRP1: Low-density lipoprotein receptor-related protein 1
MI: Myocardial infarction
MRI: Magnetic resonance imaging
oxLDL: Oxidized LDL
PDGF: Platelet-derived growth factor
PKA: Protein kinase A
SIS: Segment involvement score
SMC: Smooth muscle cells
SSS: Segment stenosis score
TG: Triglycerides
TF: Tissue factor
TNFα: Tumor necrosis factor-α
