# Supplementary material for: Reduced blood EPAC1 protein levels as a marker of severe coronary artery disease: the role of hypoxic foam cell-transformed smooth muscle cells
Source: J Transl Med. 2025 May 9;23:523. doi: 10.1186/s12967-025-06513-3 (PMC12063457; doi:10.1186/s12967-025-06513-3)
Supplement: Supplementary file 1 — Supplementary material 1 [file 12967_2025_6513_MOESM1_ESM.pptx]

## Slide 1
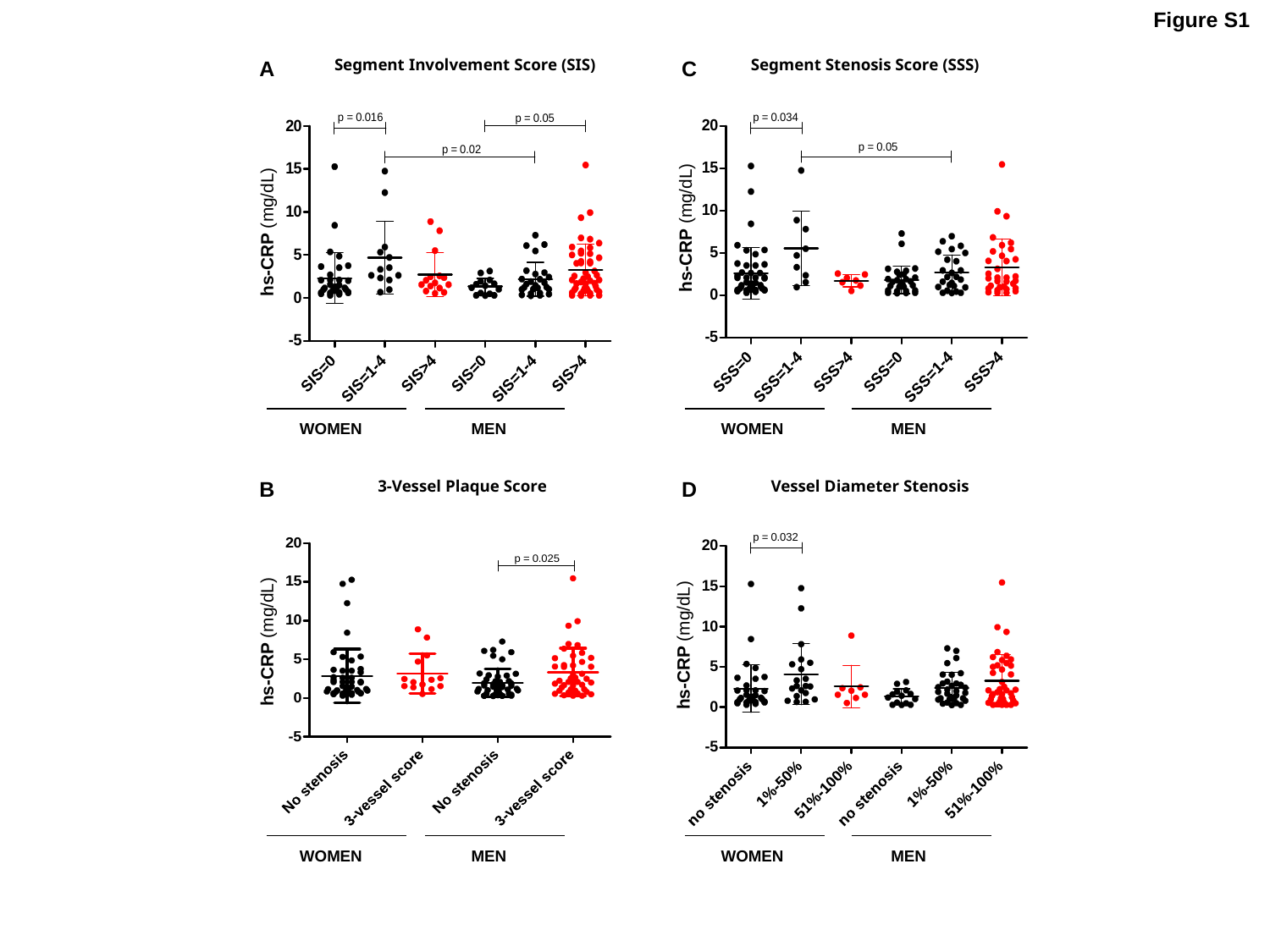

Figure S1
A
Segment Involvement Score (SIS)
C
Segment Stenosis Score (SSS)
hs-CRP (mg/dL)
hs-CRP (mg/dL)
WOMEN
MEN
WOMEN
MEN
B
3-Vessel Plaque Score
D
Vessel Diameter Stenosis
hs-CRP (mg/dL)
hs-CRP (mg/dL)
WOMEN
MEN
WOMEN
MEN

## Slide 2
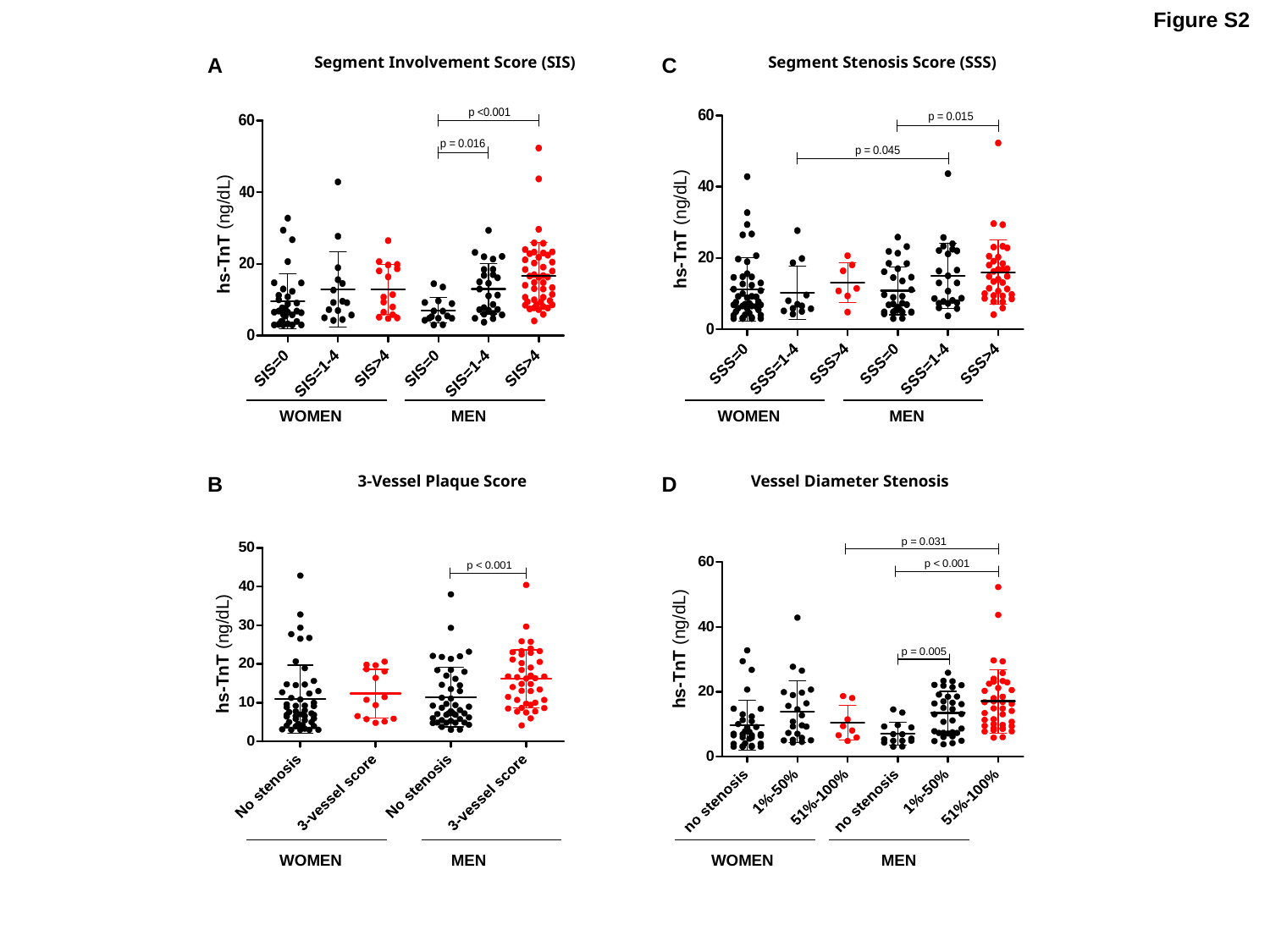

Figure S2
A
Segment Involvement Score (SIS)
C
Segment Stenosis Score (SSS)
hs-TnT (ng/dL)
hs-TnT (ng/dL)
WOMEN
MEN
WOMEN
MEN
B
3-Vessel Plaque Score
D
Vessel Diameter Stenosis
hs-TnT (ng/dL)
hs-TnT (ng/dL)
WOMEN
MEN
WOMEN
MEN

## Slide 3
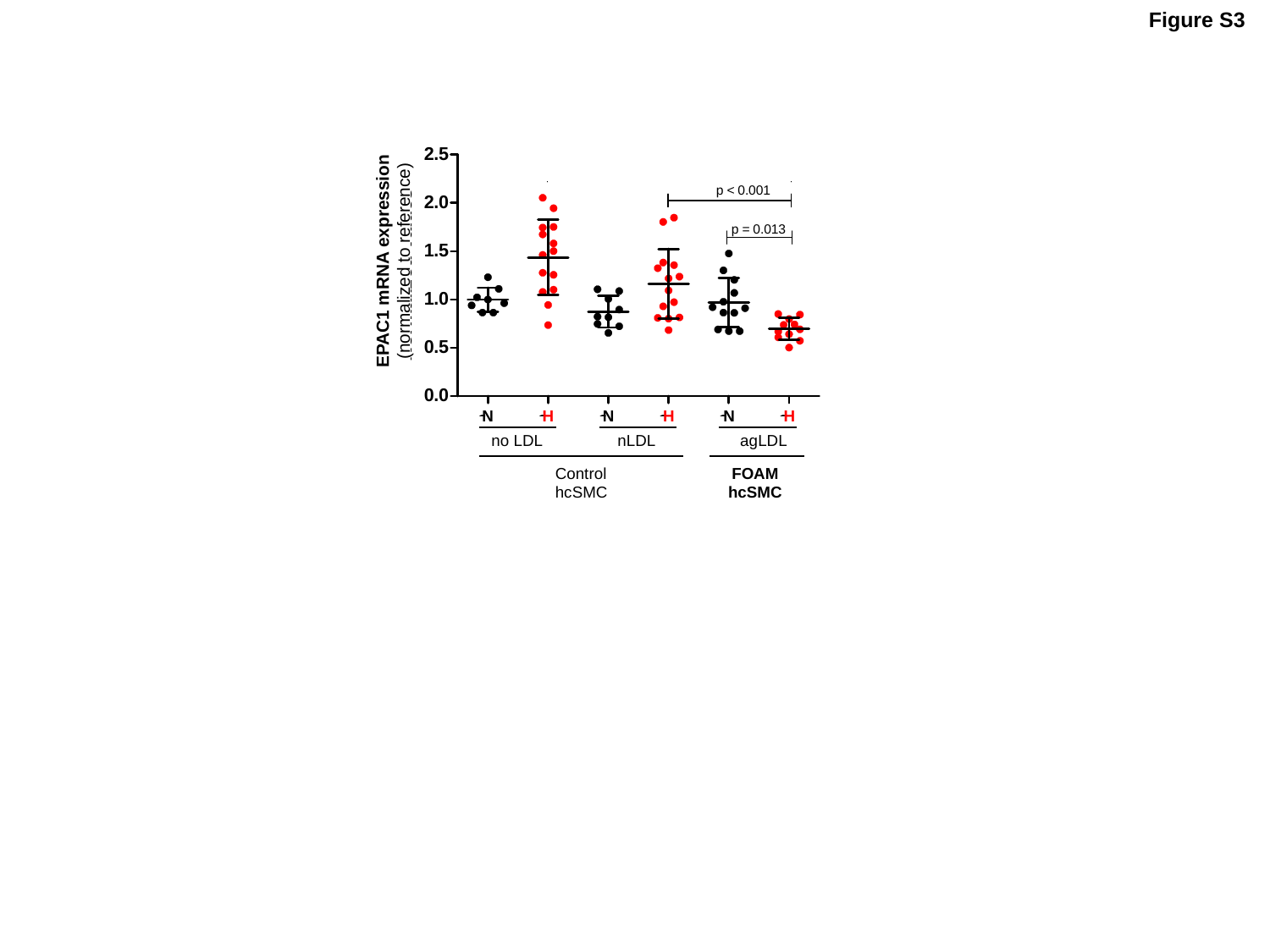

Figure S3
EPAC1 mRNA expression
(normalized to reference)
N H N H N H
no LDL
nLDL
agLDL
Control
hcSMC
FOAM
hcSMC
