# Supplementary material for: Reduced blood EPAC1 protein levels as a marker of severe coronary artery disease: the role of hypoxic foam cell-transformed smooth muscle cells
Source: J Transl Med. 2025 May 9;23:523. doi: 10.1186/s12967-025-06513-3 (PMC12063457; doi:10.1186/s12967-025-06513-3)
Supplement: Supplementary file 2 — Supplementary material 2 [file 12967_2025_6513_MOESM2_ESM.docx]

**Table S1**. Spearman correlations in all patients, CAD patients, CAD women and CAD men. CAD, coronary artery disease; SIS, segment involvement score ; SSS, segment stenosis score; hs-TnT, high-sensitivity troponin T ; hs-CRP, high-sensitivity C-reactive protein; 3v-score, 3-vessel score

| Total indivduals | Max Stenosis | SIS | SSS | 3v_Score | hs-CRP | hs-TnT | EPAC1 |
| --- | --- | --- | --- | --- | --- | --- | --- |
| Max Stenosis | 1 | 0.84314  p = 9E-56 | 0.85625  p = 3E-59 | 0.84059  p = 4E-55 | 0.18774  p = 0,02 | 0.39794  p = 5E-7 | -0.33443  p = 5E-5 |
| SIS |  | 1 | 0.80672  p = 1.4E-47 | 0.913  p = 8E-80 |  | 0.42419  p = 5.7E-8 | -0.42175  p = 2E-7 |
| SSS |  |  | 1 | 0.80309  p = 7E-47 |  | 0.31306  p = 9E-5 | -0.34648  p = 2,6E-5 |
| 3v_Score |  |  |  | 1 | 0.24474  p = 0.003 | 0.40687  p = 2E-7 | -0.47271  p = 3E-9 |
| hs-CRP |  |  |  |  | 1 | 0.27125  p = 0.0012 |  |
| Hs-TnT |  |  |  |  |  | 1 |  |
| EPAC1 |  |  |  |  |  |  | 1 |

| CAD patients | Max Stenosis | SIS | SSS | 3v_Score | hs-CRP | hs-TnT | EPAC1 |
| --- | --- | --- | --- | --- | --- | --- | --- |
| Max Stenosis | 1 | 0.59501  p = 6E-13 | 0.83265  p = 2.5E-32 | 0.53301  p = 3E-10 |  |  | -0.20515  p = 0.03318 |
| SIS |  | 1 | 0.68312  p = 6E-18 | 0.7519  p = 3E-23 |  | 0.23207  p = 0.018 | -0.34242  p = 0.0009 |
| SSS |  |  | 1 | 0.6833  p = 6E-18 |  |  | -0.25251  p = 0.008 |
| 3v_SCORE |  |  |  | 1 |  |  | -0.44832  p = 0.000001 |
| hs-CRP |  |  |  |  | 1 | 0.35745  p = 3.8E-4 |  |
| hs-TnT |  |  |  |  |  | 1 |  |
| EPAC1 |  |  |  |  |  |  | 1 |

| CAD Men | Max Stenosis | SIS | SSS | 3v_Score | hs-CRP | hs-TnT | EPAC1 |
| --- | --- | --- | --- | --- | --- | --- | --- |
| Max Stenosis | 1 | 0.637  p = 1.4E-12 | 0.779  p = 2E-21 | 0.527  p = 2E-8 |  | 0.234  p = 0.04747 |  |
| SIS |  | 1 | 0.7236  p = 2E-17 | 0.818  p = 5E-25 |  |  | -0.338  p = 0.00281 |
| SSS |  |  | 1 | 0.665  p = 6E-14 |  |  | -0.2397  p = 0.03703 |
| 3v_Score |  |  |  | 1 |  |  | -0.4326  p = 1E-4 |
| hs-CRP |  |  |  |  | 1 | 0.4314  p = 2.4E-4 |  |
| hs-TnT |  |  |  |  |  | 1 |  |
| EPAC1 |  |  |  |  |  |  | 1 |

| CAD women | Max Stenosis | SIS | SSS | 3v_score | hs-CRP | hs-TnT | EPAC1 |
| --- | --- | --- | --- | --- | --- | --- | --- |
| Max Stenosis | 1 | 0.4543  p = 0.007 | 0.8145  p = 4.6E-9 | 0.6813  p = 9.2E-6 |  |  |  |
| SIS |  | 1 | 0.4675  p = 0.0053 |  |  |  |  |
| SSS |  |  | 1 | 0.651  p = 3E-5 |  |  |  |
| 3v_Score |  |  |  | 1 |  |  | -0.4435  p=0.01101 |
| hs-CRP |  |  |  |  | 1 |  |  |
| hs-TnT |  |  |  |  |  | 1 |  |
| EPAC1 |  |  |  |  |  |  | 1 |

**Table S2. Univariate analysis of variance for EPAC1**

| **Variable** | **F** | **p** |
| --- | --- | --- |
| Sex | 2.369 | 0.126 |
| SIS | 5.311 | < 0.001 |
